# Longitudinal changes in cognition, sleep, and psychological distress following the MORE program in head and neck cancer patients undergoing chemoradiotherapy

**DOI:** 10.1038/s41598-026-52805-z

**Published:** 2026-05-23

**Authors:** Hritika D. Pai, K. Vijaya Kumar, Prasanna Mithra, Aishwariya Narasimhan, M. S. Athiyamaan, Justin Keogh, Stephen Rajan Samuel

**Affiliations:** 1 Department of Physiotherapy, Kasturba Medical College Mangalore, Manipal Academy of Higher Education, Manipal, India; 2 Department of Community Medicine, Kasturba Medical College Mangalore, Manipal Academy of Higher Education, Manipal, India; 3 Department of Radiation Oncology, Kasturba Medical College Mangalore, Manipal Academy of Higher Education, Manipal, India; 4 Faculty of Health Sciences and Medicine, Bond University, Gold Coast, Australia; 5 Mount Vernon Nazarene University, Mount Vernon, OH USA

**Keywords:** Cognition, Distress, Head and neck cancer, Multimodal exercise, Rehabilitation, Sleep, Cancer, Health care, Oncology, Psychology, Psychology

## Abstract

The purpose of this study was to evaluate changes in subjective cognitive function, sleep quality and psychological distress among head and neck cancer (HNC) patients receiving concurrent chemoradiotherapy (CRT) participating in the Multimodal Oncology Rehabilitation Exercise (MORE) program. A total of 118 HNC patients (median age 47 years, 78.8% males) participated in the supervised program, which included physical exercises, cognitive exercises and psychosocial strategies, delivered three times weekly over eight weeks. Outcomes were assessed at baseline, four, eight (discharge) and twelve weeks (follow-up) using subjective outcome measures. The linear mixed-effects models were employed to evaluate changes in outcomes over time. Results revealed significant improvements in perceived cognitive impairment (β: 3.45, *p* < 0.001), sleep (β: 2.8, *p* < 0.001), and psychological distress (β: − 2.4, *p* < 0.001) by the 12th week (follow-up). These benefits were not influenced by demographic or clinical characteristics. These findings highlight the potential of structured, multimodal exercise programs as adjuncts to supportive care for improving cognition, sleep and psychological distress among HNC patients, particularly in resource constrained settings. To the best of our knowledge, this is the first study to evaluate the longitudinal changes of such an intervention among Indian HNC patients, underscoring the need for future large-scale randomized controlled trials.

## Introduction

Advances in diagnostic and therapeutic modalities over the past decades have led to increased survival rates among head and neck cancer (HNC) patients^1^. HNC remains a major public health concern in India, accounting for nearly 26% of all cancer cases in males and 8% among females^2^. Chemoradiotherapy (CRT) remains the cornerstone of treatment for HNC, and is associated with significant declines in muscle mass, strength, as well as physical function^3^. Literature suggests that, declines in physical performance outcomes are associated with changes in cognition, sleep and psychosocial outcomes among cancer survivors, including older adults with cancer^4^.

Although cognitive impairments following CRT among HNC patients remains well-established^5,6^, structured exercise-based interventions addressing these deficits remain under-explored. Radiotherapy can cause several adverse effects to cognition, impairing memory and executive functions^1^. Meanwhile, chemotherapy causes cancer-related cognitive impairments (CRCI) via mechanisms like reduction in neurogenesis, disruption of the blood brain barrier, neuroinflammation and oxidative stress, thus impacting memory, attention and executive functions^7^. Disturbances in sleep and psychological distress are quite prevalent among HNC patients, with over 66% reporting poor sleep quality before, during and after treatment^8^. Several studies have suggested that persistent pain, xerostomia, fear of recurrence as well psychological complaints such as anxiety and depression which are highly prevalent in HNC, can further exacerbate sleep disturbances^8,9^.

Emerging evidence suggest that exercise interventions may improve cognitive functions in young and older adults^10,11^, with the ability to improve memory, attention and executive functions^12^. Systematic reviews of randomized controlled trials have highlighted the beneficial role of exercise on CRCI, particularly among breast cancer survivors followed by hematological and prostate cancer survivors, with several studies reporting improvements in self-reported cognitive functioning^13^. Although evidence specific to HNC remains sparse, emerging evidence underscores the beneficial role of exercise in mitigating cognitive decline, alongside improvements in psychological well-being and sleep quality^13,14^.

Previous studies incorporating multimodal approaches have led to preserved body composition, reduced visceral fat, improved cardiorespiratory fitness, enhanced treatment-related functional outcomes, and improved quality of life among HNC patients^15–18^. Prehabilitation studies combining exercise, nutrition, and psychological support have also shown promise^17^. Thus, these findings underscore the potential of multimodal approaches in addressing complex physical and psychosocial challenges experienced by HNC patients. Building on this evidence, the Multimodal Oncology Rehabilitation Exercise (MORE) program was developed as a structured, exercise-based intervention tailored for HNC patients undergoing concurrent CRT. The program integrates physical exercises, cognitive training (cognitive exercises and cognitive-motor tasks) and psychosocial strategies to comprehensively address multidimensional challenges experienced during and post treatment.

Despite the high burden and unique challenges faced by Indian HNC patients, there is a paucity of evidence investigating the impact of culturally tailored, structured multimodal exercise programs on cognition, sleep, and psychosocial distress in this cohort. Much of the literature examining the cognitive and psychosocial outcomes among HNC patients are drawn from the western population^19^, which differs in sociocultural dynamics and may impact effectiveness of the intervention among the Indian population. Given the sociocultural and literacy diversity among the Indian HNC cohort, we culturally adapted the MORE program to enhance accessibility, and adherence. Thus, the current study aims to analyze the longitudinal changes of the MORE program, a structured, multimodal intervention on the subjective cognitive functioning, as well as sleep and psychological distress among HNC patients undergoing concurrent CRT in a tertiary care set-up in India.

## Methods

### Study design

The current single—group longitudinal study with repeated measures design was conducted at Kasturba Medical College Hospital, Attavara, Mangalore, Karnataka, India. Ethics Approval was obtained from the Institutional Ethics Committee (IEC KMC MLR 05/2023/205). The study was also registered in the Clinical Trials Registry India (CTRI/2024/02/062366). The study was conducted abiding with ethical principles of the Declaration of Helsinki for research involving human participants.

### Participants

The study participants were deemed eligible for recruitment if they had a histologically confirmed diagnosis of HNC-TNM stages III–IVb, were 18 years or older, were scheduled to receive concurrent CRT and had an Eastern Cooperative Oncology Group-Performance Status (ECOG -PS) score of 0–2. Stages III–IVb were selected as these patients typically undergo concurrent CRT and experience a higher treatment-related symptom burden thereby necessitating a need for rehabilitation interventions^20^. All participants were scheduled for concurrent CRT at the time of enrollment (baseline) and received active treatment throughout the supervised inpatient phase (baseline to week 8), supported by a strong body of evidence in cancer-rehabilitation studies^20,21^. The exclusion criteria comprised of significant comorbidities restricting participation, non-ambulatory status, and/or patients scheduled for palliative treatments.

The sample size for this study was calculated based on results from an unpublished pilot study, which suggested a minimally interesting effect size (δ) of 0.35 for the primary outcome. Accounting for a potential dropout rate of 20%, a total of 106 participants were determined to be necessary to achieve 90% power with a two-tailed α of 0.05. These calculations were performed using *Jamovi s*oftware (version 2.3.26), J power module. This effect size aligns with small-to-moderate changes reported in previous exercise oncology studies incorporating patient-reported outcomes^13,22,23^.

HNC patients were recruited from May 2023 to February 2025, following referrals from radiation oncologists. Prior to recruitment, an informed consent form was obtained from each patient who met the inclusion criteria. A single-group repeated measures design was adopted for the current longitudinal study as logistical constraints in delivering a multimodal inpatient rehabilitation intervention, ethical considerations like withholding access to exercise-based rehabilitation in a motivated and vulnerable patient population made randomization unfeasible. Additionally, a longitudinal repeated-measures design allowed assessment of outcomes across multiple timepoints i.e., baseline, during and after active treatment, thereby capturing within-subject changes over time, by employing the linear mixed-effects models.

### Medical management

The medical regime consisted of a definitive dose of CRT with 70 Grays of radiation over 35 fractions for non-operated patients and adjuvant dose of CRT comprising of 66 Grays of radiation over 33 fractions post operatively. Concurrent chemotherapy was delivered with Cisplatin at 40 mg/m^2^ weekly or Carboplatin under AUC 2 or Cisplatin at 30 mg/m^2^ with Tab. Capecitabine 625 mg/m^2^ twice daily.

### Recruitment and intervention

A total of 118 HNC patients were recruited into the MORE program, an eight-week inpatient exercise-based intervention delivered during their continuous hospital admission for concurrent chemoradiotherapy at Kasturba Medical College Hospital. The MORE program was developed by two trained physical therapists from the Department of Physiotherapy, Kasturba Medical College Mangalore, India and the pilot feasibility study was conducted at the same centre. The structure of the MORE program was based on previous evidence supporting the integration of multimodal rehabilitation programs in HNC populations^15–17^.

Patients remained hospitalized for the duration of the anti-cancer treatment i.e., 8 weeks and participated in the MORE program which comprised of cognitive, and physical exercise-based interventions along with psychosocial strategies (relaxation exercises and psychoeducation) delivered by trained physical therapists in Kannada language (see Table 1). During the inpatient phase the intervention was delivered three times weekly on non-chemoradiotherapy days to minimize patient burden. Each session lasted approximately 45 min, and included 20 min of cognitive exercises, 15 min of physical exercises, and 10 min of psychoeducational strategies. Sessions were planned in coordination with the oncology team.

In our study, the term culturally tailored refers to the integration of the local language, pictorial instructions, and adaptation of the exercise regimes to patients’ literacy levels, ensuring accessibility and addressing barriers unique to the Indian scenario while actively involving family members and caregivers.

**Fig. 1:**
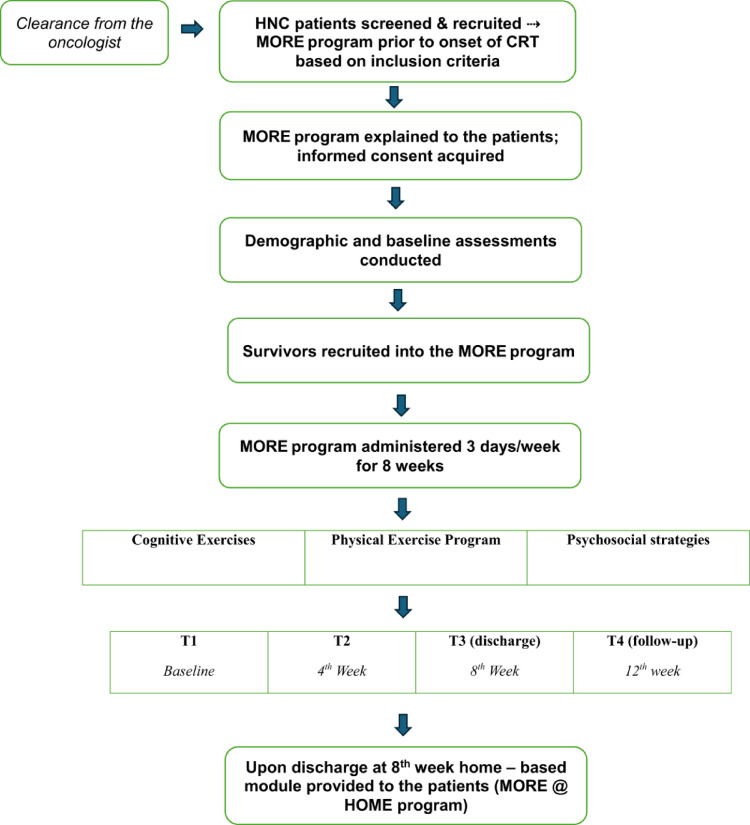
Research timeline of the Multimodal Oncology Rehabilitation Exercise (MORE) program.

To bridge the inpatient and post-discharge phases, the MORE @ HOME module was introduced at week 8 before hospital discharge. Patients were instructed to perform the exercises 3 times per week for 4 weeks to ensure continuity of the intervention at home. This home-based module consisted of pictorial description of physical exercises, cognitive exercise, cognitive-motor tasks, and breathing exercises lasting approximately 20–30 min per day. The module was delivered in Kannada, with an activity log to track adherence at home and the patients were reassessed at week 12 (follow-up) promoting sustained engagement in the exercise regime and long-term rehabilitation for HNC patients recruited in our study. The timeline for assessments and interventions is detailed in Fig. 1.

The cognitive exercises were curated to comprehensively target 4 cognitive domains predominantly affected in HNC patients undergoing concurrent CRT i.e., attention, memory, learning, and executive functions through progressively challenging cognitive exercises including mazes and problem solving tasks^24^. The difficulty levels were adjusted weekly, with the intent of supporting cognitive improvement and neuroplasticity which is in line with a growing body of evidence i.e., repetitive and progressively challenging tasks promote improvements in subjective and objective cognitive functioning among cancer survivors and older adults^25,26^. In addition, cognitive-motor tasks were incorporated alongside physical exercises; for instance backward counts during resistance exercises of the upper-limb, these tasks were targeted to engage working memory, executive functions and also enhance dual-task performance by engaging the prefrontal and motor cortex simultaneously^27,28^.

The psychosocial strategies included relaxation exercises i.e., diaphragmatic breathing and Jacobson’s progressive muscle relaxation strategies to target symptoms of psychological distress (depression and anxiety) and sleep disturbances. Literature suggests that these strategies activate a parasympathetic response, decrease psychological arousal and have been shown to significantly reduce depressive and anxious thoughts as well as improve sleep quality^29,30^. The psychoeducational program emphasised the benefits of exercise pre, during and post cancer treatment. The program was delivered by a trained therapist in the local language. The sessions specifically highlighted that exercise during cancer treatment has a broad range of benefits such as counteracting fatigue, muscle wasting, improving mobility, physical function, cognition and sleep quality as well as being able to reduce depressive and anxious symptoms^13,31,32^. Patients and their caregivers were educated regarding the barriers and misconceptions regarding exercise i.e., addressing fears, lack of energy, and limited access to resources and informed that exercise is safe and can be performed in the comfort of their homes. Family members or caregivers were encouraged to participate in daily exercises, and strategies were provided for incorporating exercises into household routines. Given the high prevalence of tobacco and areca nut chewing among Indian HNC patients, these sessions also included counselling regarding the risks associated with these habits, impact of continued use on recovery and long-term health outcomes. Thus, these sessions not only aimed to promote exercise as an adjunctive therapy among HNC patients, but also included strategies to address chewing-related barriers to rehabilitation, and thus improve overall treatment-related outcomes.

**Table 1:** The Multimodal Oncology Rehabilitation Exercise (MORE) program: Training Protocol.

Domain	Component	Frequency	Intensity	Time	Type
Cognitive	1. Cognitive Exercises 2. #Cognitive-motor tasks (incorporated along with the physical domain)	3 days/week for 8 weeks	Moderate. Complexity of the task was gradually increased (e.g., simple puzzles and mazes complex to activities)	20 min/ session	Aimed to address memory, learning, and executive function **Cognitive Exercises**: a. **Puzzles** b. **Mazes** Each activity was performed 3 times/week for 8 weeks. Patients maintained a log to record the time taken to complete each task, with a decrease in completion time indicating better adherence to the program as sessions progressed.
Physical	Walking + Resistance training program with cognitive-motor strategies	3 days/week for 8 weeks	RPE 3–5/10	15 min/ session	**Walking program** → progression each week (e.g., normal pace, brisk walking, task based walking, stair climbing). **Resistance exercises** upper-limb and lower-limb strength using resistance bands or household objects (e.g., water bottles). **Jaw & neck exercises**: range of motion, isometric strengthening and stretching exercises as well as ice - cream stick manoeuvres for trismus*. Progression: Gradual increase in intensity, resistance, and task complexity as the weeks advance, tailored to the comfort level of the patients. **#Cognitive-motor tasks**: Week 1: Count backwards 10 − 0 Week 2: Reciting the days of the week backwards (e.g., Sunday, Saturday,…Monday.) Week 3: Alternate between numbers and days of the week (e.g., Monday-1, Tuesday-2 and so on) Week 4: Count using multiples of 2’s and 3’s (e.g., 2, 4, 6. and 3, 6, 9…)
Psychosocial strategies	Relaxation Strategies	3 days/week for 8 weeks	Based on participant comfort	10 min/ session	**Diaphragmatic breathing** exercises and standard procedure for Jacobson’s progressive muscle relaxation technique.
	Psychoeducation				Education on the benefits of exercise pre, during and post cancer treatment for patients and caregivers in their native language

The MORE program was delivered three times per week during the inpatient phase (baseline to week 8). *The use of ice cream sticks to address trismus in a specific group of patients proved to be a cost-effective strategy. #Cognitive-motor tasks were incorporated in the physical exercise domain.

### Outcome measures

Patient-reported outcome measures were used for the present study to evaluate cognition, sleep and psychological distress. The Functional Assessment of Cancer Therapy-Cognitive Function (FACT-Cog, version 3)^33^, evaluates perceived cognitive impairments (PCI), abilities (PCA), their impact on quality of life (QOL), and comments from others (OTH); with higher scores indicating better cognitive functions. The Cronbach’s α coefficient for FACT-Cog for the individual subdomains include PCI = 0.97, PCA = 0.93, QOL = 0.92, OTH = 0.92^34^. Subjective sleep quality was evaluated using Pittsburgh Sleep Quality Index (PSQI), a widely used tool, that generates a global score ranging from 0 to 21, with higher scores indicating poorer sleep quality^35^. The PSQI demonstrated moderate to high internal consistency across groups and correlations between global and component scores (Cronbach’s α = 0.80)^36^. The Patient Health Questionnaire-4 (PHQ-4) was used to assess psychological distress, including symptoms of depression and anxiety, demonstrating a high internal consistency (Cronbach’s α = 0.92)^37^. This tool combines scores of four items to generate a composite score ranging from 0 to 12, with higher values denoting greater levels of distress^37^. For the present study, the Kannada versions of the FACT-Cog and PSQI were administered to the patients enrolled in the MORE program with permissions from the developers of the tool^33,35^. All three outcome measures were administered at four time points i.e., baseline, 4^th^ week, 8^th^ week (discharge) and 12^th^ week (follow-up). Adherence to the intervention during the 8-week (inpatient stay) period was monitored by calculating the total number of sessions attended out of 24 sessions. At the 12^th^ week follow-up, exercise adherence was further evaluated using the Exercise Adherence Rating Scale-Kannada version (EARS-Kn)^38^.

### Statistical analysis

Data analysis was performed using the IBM SPSS software (Version 29.0, IBM Corp., Armonk, NY, USA). Demographics and clinical characteristics were summarised for baseline data using descriptive statistics. The normality of all continuous data was assessed using the Shapiro-Wilk test, which revealed that the data was not normally distributed, therefore these variables were reported as median and interquartile range (IQR), categorical variables were reported as counts and percentages. Changes in patient-reported outcomes were evaluated over time using the linear mixed-effects models, with participants considered as random effects to account for within-subject correlations and time as a fixed effect, along with baseline characteristics (age, gender, stage of cancer, and surgical status) and lifestyle behaviors (smoking and alcohol consumption) as covariates. Individual models considered time and demographic characteristics as fixed effect. As none of the examined covariates demonstrated statistically significant associations, multivariable adjusted models were not constructed. Additionally, in our study the proportion of missing data was low (< 5%), and complete-case analysis was conducted to handle missing data within the linear mixed-effect model framework. Beta coefficients (β), 95% confidence intervals (CI), and p-values were reported for fixed effects. Statistical significance was set at a two-tailed p-value of < 0.05.

## Results

In the current study we recruited a total of 133 HNC patients. Fifteen patients (11.3%) were lost to follow-up, or died during the course of treatment or transitioned to palliative care. Thus, 118 patients were included in the final study and analysis, exceeding the calculated sample size. The median age of the patients was 47 years, with predominantly male patients (78.8%). Tobacco chewing was highly prevalent among patients (94.1%), while 38.1% were smokers. The oral cavity (62.7%) was the most common primary tumor site followed by the pharynx (24.6%), and the larynx (10.2%). The majority of patients (53.4%) presented with stage IVa of the disease. Chemotherapy regimens involved Cisplatin (46.2%), followed by a combination of Cisplatin + Capcecitabine (22.2%). Patients received a median dose of 33 fractions of radiation over 8 weeks. Demographic details and baseline characteristics for the 118 patients are provided in Table 2.

Adherence to the MORE program was relatively high, with patients attending an average of 18 ± 2.77 sessions of 24 sessions during the inpatient phase (baseline to 8 weeks)^39^. Adherence at week 12 (follow-up) was assessed using the Exercise Adherence Rating Scale-Kannada version (EARS-Kn); scores ranging from 0 to 24, with higher scores indicating greater adherence. The median score of 19 of 24 in the current study corresponds to approximately 79% of the maximum score and exceeds the cut-off of 17 (95.83% sensitivity, 80% specificity), indicating a high adherence to the program during the post-discharge phase^38^.

**Table 2:** Baseline Demographics, Clinical Characteristics, and Outcomes Assessed (*N* = 118).

Characteristic (*N* = 118)		Reported as mean ± SD/median (IQR)/Counts (%) where applicable.			
		Counts	Percentage (%)	Median	Interquartile Range (IQR)
Demographics					
Age (years)		–	–	47	43.25 - 54
Gender	Male	93	78.8	–	–
	Female	25	21.2	–	–
Tobacco Chewing	(% Chewers)	111	94.1	–	–
Tobacco Smoking (Beedi/Bidi smoking)		45	38.1	–	–
Alcohol consumption		37	31.4	–	–
**Clinical Characteristics**					
**Site of Tumor**					
Oral Cavity		74	62.7	–	–
Pharynx		29	24.6	–	–
Larynx		12	10.2	–	–
Nasal cavity & paranasal sinuses		1	0.8	–	–
Others		2	1.7	–	–
**Stage of tumor**	III	29	24.6		
	IVa	63	53.4		
	IVb	26	22		
**Radiation Fractions (#)**		–	–	33	33 - 35
**Chemotherapy Drugs**	Cisplatin	54	46.2	–	–
	Cisplatin + Capecitabine	26	22.2	–	–
	Carboplatin	19	16.2	–	–
	No chemotherapy	18	15.4	–	–
**ECOG-PS score**	0	15	12.7	–	–
	1	96	81.4	–	–
	2	7	5.9	–	–
**Subjective Cognitive Performance**					
Cog - Perceived cognitive abilities		–	–	23	22 - 25
Cog - Perceived cognitive impairments		–	–	60.5	56 - 65
Cog - Comments from others		–	–	14	12 - 15
Cog - Impact on quality of Life		–	–	12	12 - 14
**Sleep**					
Pittsburgh Sleep Quality Index (PSQI)		–	–	9	8 - 11
**Psychological Distress**					
Patient Health Questionnaire-4 (PHQ-4)		–	–	6	5 - 7

Significant changes in subjective cognitive scores were observed over time. By the 4th week, the adjusted mean for the Cog-PCI significantly decreased by 1.78 points (95% CI: -2.02 to -1.55, *p* < 0.001) relative to baseline. In contrast, significant improvements at the 8th week (β = +1.01; 95% CI: 0.78 to 1.25, *p* < 0.001, and at follow-up (β = +3.45; 95% CI: 3.22 to 3.69, *p* < 0.001) were observed compared to baseline. Similar trends were observed for perceived cognitive abilities (PCA), comments from others (OTH) and impact on quality of life (QOL) subdomains of the FACT-Cog; with significant improvements evident at 8 and 12 weeks (*p* < 0.001). However, none of the demographic or clinical covariates (age, gender, stage of tumor, surgical status, tobacco/alcohol consumption or chemotherapy status) included in the models demonstrated a statistically significant association with the magnitude of change in the subjective cognitive scores over time. The results of the univariate analysis of subjective cognitive outcomes over time are presented in Table 3.

**Table 3:** Univariate Analysis of Factors associated with subjective cognitive outcomes among HNC patients using linear mixed effects model.

Variables	Cog-PCI		Cog-QOL		Cog-OTH		Cog - PCA	
	β (95% CI)	*p*-value	β (95% CI)	*p* - value	β (95% CI)	*p* - value	β (95% CI)	*p* - value
**Time**								
Baseline	**REF**		**REF**		**REF**		**REF**	
4 weeks	−1.78 (−2.02, −1.55)	< 0.001*	−0.50 (−0.66, −0.34)	< 0.001*	−0.48 (−0.68, −0.29)	< 0.001*	−0.75 (−1.01, −0.49)	< 0.001*
8 weeks	1.01 (0.78, 1.25)	< 0.001*	0.32 (0.16, 0.48)	< 0.001*	0.57 (0.37, 0.76)	< 0.001*	1.36 (1.10, 1.62)	< 0.001*
12 weeks	3.45 (3.22, 3.69)	< 0.001*	0.68 (0.52, 0.84)	0.264	1.49 (1.30, 1.69)	< 0.001*	3.16 (2.90, 3.42)	< 0.001*
**Age**	0.08 (−0.08, 0.25)	0.317	0.02 (−0.02, 0.07)	0.371	0.02 (−0.01, 0.05)	0.205	0.02 (−0.008, 0.06)	0.134
**Gender**								
Female	**REF**		**REF**		**REF**		**REF**	
Male	−0.46 (−3.86, 2.94)	0.788	−0.09 (−1.11, 0.92)	0.853	−0.11 (−0.78, 0.55)	0.729	0.06 (−0.64, 0.77)	0.867
**Stage**								
Stage III	**REF**		**REF**		**REF**		**REF**	
Stage IVa	1.75 (−2.21, 5.71)	0.384	−0.78 (−0.40, 1.98)	0.195	0.45 (−0.32, 1.24)	0.25	0.46 (−0.37, 1.31)	0.277
Stage IVb	−2.8 (−6.13, 0.45)	0.09	−0.42 (−1.41, 0.56)	0.397	−0.40 (−1.05, 0.24)	0.224	−0.07 (−0.78, 0.62)	0.83
**Tobacco Consumption**								
No	**REF**		**REF**		**REF**		**REF**	
Yes	−1.32 (−10.16, 7.50)	0.766	0.49 (−3.13, 2.13)	0.709	−0.72 (−2.46, 1.01)	0.411	−0.91 (−2.74, 0.92)	0.327
**Alcohol Consumption**								
No	**REF**		**REF**		**REF**		**REF**	
Yes	−1.72 (−4.70, 1.25)	0.254	−0.02 (−0.91, 0.87)	0.962	−0.30 (−0.89, 0.28)	0.303	−0.17 (−0.80, 0.44)	0.574
**Surgery**								
Non - Operated	**REF**		**REF**		**REF**		**REF**	
Operated	2.09 (−1.39, 5.58)	0.237	0.83 (−0.20, 1.86)	0.115	0.28 (−0.40, 0.97)	0.411	0.12 (−0.60, 0.85)	0.73
**Chemotherapy Status**								
Received	**REF**		**REF**		**REF**		**REF**	
Not-Received	0.82 (−3.05, 4.71)	0.673	0.17 (−0.98, 1.33)	0.761	−0.07 (−0.83, 0.69)	0.85	−0.37 (−1.18, 0.42)	0.353

β represents “estimate” indicating the fixed effect coefficients, representing the change in the dependent variable associated with each independent variable. *Statistically significant *p* < 0.05; CI: Confidence interval. Cognition was assessed using the Functional Assessment of Cancer Therapy-Cognitive Function (FACT - Cog) version 3.

Global PSQI scores did not differ significantly from baseline at 4 weeks (β = −0.22, 95% CI: −0.71 to 0.27, *p* = 0.383). However, statistically significant reductions were observed at the 8th week (β = −1.34, 95% CI: −1.84 to −0.85, p = < 0.001) and 12th week (β = −2.81, 95% CI: −3.03 to −2.31, *p* < 0.001) relative to baseline. No significant main effects over time were observed for demographic or clinical covariates.

Psychological distress measured using the PHQ-4 scale at the 4th week did not differ significantly from baseline (β = 0.10, 95% CI: −0.12 to 0.34, *p* = 0.371). However, statistically significant improvements were observed at discharge i.e., week 8 (β = −0.95, 95% CI: −1.19 to −0.72, *p* < 0.001). These reductions were maintained at follow-up (β = −2.39, 95% CI: −2.63 to −2.16, *p* < 0.001) compared to the baseline. Although the PHQ-4 lacks oncology specific (Minimal Clinically Important Differences) MCIDs, evidence from general population suggests a 2–3 point change may be meaningful across time^40^. No statistically significant associations were observed between demographic, clinical factors and changes in psychological distress. Univariate analysis of the changes in the sleep quality and psychosocial distress over time are presented in Table 4.

**Table 4:** Univariate Analysis of Factors associated with sleep and psychological distress among HNC patients using linear mixed effects model.

Variables	Pittsburgh Sleep Quality Index (PSQI) scores		Patient Health Questionnaire-4 (PHQ-4)	
	β (95% CI)	*p*-value	β (95% CI)	*p* - value
**Time**				
Baseline	**REF**		**REF**	
4 weeks	−0.22 (−0.71, 0.27)	0.383	0.10 (−0.12, 0.34)	0.371
8 weeks	−1.34 (−1.84, −0.85)	< 0.001*	−0.95 (−1.19, −0.72)	< 0.001*
12 weeks	−2.81 (−3.03, −2.31)	< 0.001*	−2.39 (−2.63, −2.16)	< 0.001*
**Age**	0.01 (−0.02, 0.05)	0.578	0.00 (−0.01, 0.02)	0.668
**Gender**				
Female	**REF**		**REF**	
Male	0.68 (−0.11, 1.47)	0.092	−0.09 (−0.54, 0.36)	0.693
**Stage**				
Stage III	**REF**		**REF**	
Stage IVa	−0.76 (−1.71, 0.19)	0.115	−0.08 (−0.62, 0.45)	0.757
Stage IVb	−0.71 (−1.50, 0.08)	0.078		
**Tobacco Consumption**				
No	**REF**		**REF**	
Yes	0.00 (−0.208, 2.08)	0.999	0.93 (−0.22, 2.10)	0.114
**Alcohol Consumption**				
No	**REF**		**REF**	
Yes	−0.39 (−1.09, 0.31)	0.274	−0.25 (−0.64, 0.14)	0.21
**Surgical Status**				
Non-Operated	**REF**		**REF**	
Operated	−0.31 (−1.14, 0.51)	0.451	0.01 (−0.45, 0.47)	0.966
**Chemotherapy Status**				
Received	**REF**		**REF**	
Not-Received	0.002 (−0.91, 0.92)	0.996	−0.37 (−0.88, 0.13)	0.15

β represents “estimate” indicating the fixed effect coefficients, representing the change in the dependent variable associated with each independent variable.

*Statistically significant *p* < 0.05; CI: Confidence interval. Sleep quality was assessed using the Pittsburgh Sleep Quality Index (PSQI), and depression and anxiety was assessed using the PHQ − 4 scale.

## Discussion

The present study evaluated longitudinal changes of the Multimodal Oncology Rehabilitation Exercise (MORE) program on subjective cognitive symptoms, sleep quality and psychological distress (depression and anxiety) using patient-reported outcome measures adapted to the native language among HNC patients undergoing concurrent chemoradiotherapy (CRT) in a tertiary care center in India. Unlike various multimodal exercise-based interventions conducted predominantly among western and breast cancer populations^41^, to the best of our knowledge, our study is the first to investigate a culturally tailored multimodal exercise program on the aforementioned outcomes among Indian HNC patients, a population with a high disease burden.

The MORE program targeted physical, cognitive and psychosocial domains using minimal equipment (e.g., resistance bands, household objects) and was delivered during routine hospitalization for chemoradiation using existing institutional resources, without requiring additional out-of-pocket expenses for patients. The program was delivered in Kannada (local) language, and constituted a home-based module provided at discharge (8th week). Importantly, during the inpatient phase the exercise intervention was scheduled on non-chemoradiotherapy (CRT) days to avoid interference with the medical treatments and ensure feasibility during active anti-cancer treatments. India has one of the world’s highest age-standardized incidence and mortality rates for HNC, with most patients presenting at advanced stages and facing poor treatment outcomes^2^, thus these findings highlight the potential role of structured multimodal exercise-based programs in mitigating treatment-related side-effects.

The results of our study found improvements in all domains of the FACT-Cog (Kannada version), including perceived cognitive impairment (Cog-PCI, *p* < 0.001), perceived cognitive abilities (Cog-PCA, *p* < 0.001), impact on quality of life (Cog-QOL, *p* < 0.001), and comments from others (Cog-OTH, *p* < 0.001), from baseline to 12-week (follow-up). Our data revealed an initial decline during the first four weeks, with significant improvements at the 8^th^ (discharge) and 12^th^ week. Similar trends have been observed among other cancer cohorts, with early cognitive decline attributed to acute effects of cancer treatment, fatigue and inflammation^42^. These results are consistent with recent studies that reported similar improvements in FACT-Cog subdomains following interventions such as yoga, aerobic exercise and/or combined exercise among cancer patients^43,44^. A recent meta-analysis by Pinheriro et al., reported standardized mean differences (SMD) of 0.43 for attention and working memory (*p* < 0.001) and 0.95 (*p* = 0.003) for perceived cognitive abilities among breast cancer survivors undergoing exercise interventions; aligning with the findings of our study^43^. For the FACT-Cog, breast cancer studies have estimated minimal clinically important differences (MCIDs) of 6.9–10.6 points on the total score and 4.7–7.2% change using anchor-based distribution methods. The observed changes over time in our study appear substantial but fall short of total-score MCID anchors and lack HNC specific benchmarks^45^.

Sleep disturbance is one of the most common and distressing symptoms experienced across all tumor types, which in turn affects quality of life and treatment-related outcomes^46^. It can negatively impact health-related quality of life including physical and psychological functioning^47,48^. Evidence suggests that exercise can improve sleep by reducing inflammation and fatigue, regulating melatonin and cortisol rhythms and enhancing mood^22^. A significant reduction in global PSQI scores were observed in our study by the 8^th^week (β = −1.34, *p* < 0.001) and 12th week ((β = − 2.81, *p* < 0.001), indicating clinically meaningful reductions in subjective sleep quality. For PSQI, evidence from oncology and sleep intervention studies have interpreted changes of 2–3 points on the PSQI global score as clinically meaningful^49,50^; the observed reduction in our study at 12^th^ week approaches this threshold. Our findings also align with a recent meta-analysis that demonstrated significant exercise-related improvements in sleep quality assessed with the PSQI (*p* = 0.008) among cancer patients^22^.

Patients with cancer often report high levels of depression and anxiety, with the HNC cohort being particularly vulnerable due to physical challenges such as difficulty in speech, swallowing, breathing, and xerostomia which affects their overall quality of life^51^. Emotional challenges such as treatment-induced appearance changes further exacerbate this burden^52,53^. These factors underscore the need for multimodal exercise programs which holistically target both the physical and psychological distress caused by cancer and its treatments. Patients enrolled in the MORE program experienced statistically significant reductions in psychological distress, with lower PHQ-4 scores by the 8^th^ week (β = − 0.95, *p* < 0.001) and 12^th^ week (β = − 2.39, *p* < 0.001). These reductions are consistent with a recent meta-analyses that reported significant exercise-based reductions in depression (SMD = −0.53; *p* < 0.001) and anxiety (SMD = −0.39; *p* < 0.001) across diverse cancer populations^23^.

Several underlying mechanisms can be responsible for the significant improvements seen across multiple outcomes in our study. A recent meta-analyses suggested that structured exercise programs i.e., aerobic training and/or resistance training are linked with improvements in several cognitive domains including attention, working memory, executive functions, as well as self-reported cognitive abilities. This underscores the ability of exercise as a non-pharmacological intervention to mitigate CRCI^43^. Evidence suggest that short-term exercise can help reduce fatigue and improve sleep by modulating the hypothalamic-pituitary-adrenal axis, boost skeletal muscle protein synthesis, and thus realign circadian rhythms^54^. A growing body of evidence suggests that acute bouts of structured exercise can yield clinically meaningful improvements in sleep, cognition, as well as mood among cancer survivors^22,23,43^. These findings underscore the importance of integrating structured multimodal interventions in cancer rehabilitation to improve various cancer-related adverse events.

### Limitations

This study has several limitations and requires consideration while interpreting the results and implications for future studies. Firstly, the study was not a randomized controlled trial and lacked a control group, hence we cannot ascertain that the observed improvements were due to the intervention itself, despite the tendency for these outcomes to decline among HNC patients during concurrent CRT^22,24^. Secondly, all outcomes were assessed using self-reported outcome measures, which may introduce a subjective response bias. Although our study captured patients perceived symptom burden using these outcome measures, future studies incorporating objective assessments, such as neuropsychological tests and physiological measures would further strengthen the evidence base. While many of the observed changes in scores over time appear substantial, no MCIDs have been formally established for the Kannada versions. This represents an important limitation requiring future validation studies in South Asian HNC cohorts. Thirdly, the single-center, inpatient design, and the presence of motivated HNC patients may limit the generalizability to a broader cohort of HNC patients, where several barriers to participation and follow-up times may widely differ. Furthermore, the follow-up duration of 12-weeks, limits the assessments of long-term or sustained effects.

## Conclusion

In conclusion, the burden of cognitive impairment, sleep disturbances, and psychological distress remains significant among HNC patients undergoing concurrent chemoradiotherapy. The MORE program, a structured, culturally adapted intervention, demonstrated high adherence and was associated with improvements across these domains during active anti-cancer treatment and follow-up periods. Hence, these findings support a need for further evaluation of structured, culturally adapted multimodal exercise-based interventions, such as the MORE program within oncology care. Future studies should prioritize randomized controlled trials, larger sample sizes, and extended follow-up periods.

## Data Availability

The datasets generated during and/or analyzed during the current study are not publicly available due to patient privacy but are available from the authors on reasonable request.

## References

[1] Pruijssen, J. T. et al. Long-term cognitive, psychosocial, and neurovascular complications of unilateral head and neck irradiation in young to middle-aged adults. *BMC Cancer* **22**, 244 (2022). 35248013 10.1186/s12885-022-09295-9 PMC8897732

[2] Bagal, S. et al. Head and neck cancer burden in India: An analysis from published data of 37 population-based cancer registries. *Ecancermedicalscience* **17**, 1603 (2023). 37799939 10.3332/ecancer.2023.1603 PMC10550331

[3] Chauhan, N. S., Samuel, S. R., Meenar, N., Saxena, P. P. & Keogh, J. W. L. Sarcopenia in male patients with head and neck cancer receiving chemoradiotherapy: A longitudinal pilot study. *PeerJ* **8**, e8617 (2020). 32149024 10.7717/peerj.8617 PMC7049254

[4] Loh, K. P. et al. Associations of sleep disturbance with physical function and cognition in older adults with cancer. *Support Care Cancer* **25**, 3161–3169 (2017). 28455547 10.1007/s00520-017-3724-6 PMC5660663

[5] Williams, A. M. et al. Association between cognitive function and quality of life in patients with head and neck cancer. *JAMA Otolaryngol. Head Neck Surg.* **143**, 1228–1235 (2017). 29121151 10.1001/jamaoto.2017.2014 PMC5824302

[6] Wickborn, K. et al. Timeline of cognitive impairments after radiotherapy for head and neck cancer: A review. *ctRO* 0, (2024). 10.1016/j.ctro.2024.100890 PMC11847131 39991091

[7] Demos-Davies, K., Lawrence, J. & Seelig, D. Cancer related cognitive impairment: A downside of cancer treatment. *Front. Oncol.* **14**, 1387251 (2024). 38715789 10.3389/fonc.2024.1387251 PMC11074410

[8] Santoso, A. M. M. et al. Prevalence of sleep disturbances among head and neck cancer patients: A systematic review and meta-analysis. *Sleep Med. Rev.* **47**, 62–73 (2019). 31351357 10.1016/j.smrv.2019.06.003

[9] Krebber, A.-M., Jansen, F., Cuijpers, P., Leemans, C. R. & Verdonck-de Leeuw, I. M. Screening for psychological distress in follow-up care to identify head and neck cancer patients with untreated distress. *Support Care Cancer* **24**, 2541–2548 (2016). 26694718 10.1007/s00520-015-3053-6 PMC4846709

[10] Lista, I. & Sorrentino, G. Biological mechanisms of physical activity in preventing cognitive decline. *Cell. Mol. Neurobiol.* **30**, 493–503 (2010). 20041290 10.1007/s10571-009-9488-x PMC11498799

[11] Fernandes, J., Arida, R. M. & Gomez-Pinilla, F. Physical exercise as an epigenetic modulator of brain plasticity and cognition. *Neurosci. Biobehav. Rev.* **80**, 443–456 (2017). 28666827 10.1016/j.neubiorev.2017.06.012 PMC5705447

[12] Mandolesi, L. et al. Effects of physical exercise on cognitive functioning and wellbeing: Biological and psychological benefits. *Front. Psychol.* **9**, 509 (2018). 29755380 10.3389/fpsyg.2018.00509 PMC5934999

[13] Campbell, K. L. et al. The effect of exercise on cancer-related cognitive impairment and applications for physical therapy: Systematic review of randomized controlled trials. *Phys. Ther.* **100**, 523–542 (2020). 32065236 10.1093/ptj/pzz090 PMC8559683

[14] Alnawwar, M. A. et al. The effect of physical activity on sleep quality and sleep disorder: A systematic review. *Cureus* **15**, e43595 (2025). 10.7759/cureus.43595 PMC10503965 37719583

[15] Yen, C.-J. et al. Multimodal exercise ameliorates exercise responses and body composition in head and neck cancer patients receiving chemotherapy. *Support. Care Cancer* **27**, 4687–4695 (2019). 30949831 10.1007/s00520-019-04786-1

[16] Cnossen, I. C. et al. Multimodal guided self-help exercise program to prevent speech, swallowing, and shoulder problems among head and neck cancer patients: A feasibility study. *J. Med. Internet Res.* **16**, e74 (2014). 24610383 10.2196/jmir.2990 PMC3961811

[17] Groen, L. C. B. et al. Multimodal Prehabilitation in Head and Neck Cancer Patients Undergoing Surgery: A Feasibility Study. *J. Hum. Nutr. Dietetics*. **38**, e70047 (2025). 10.1111/jhn.70047 PMC11950714 40150935

[18] Song, J. et al. Effects of multimodal exercise on health-related physical fitness and quality of life in patients with nasopharyngeal carcinoma during radiotherapy. *Pain Manag. Nurs.* **24**, 650–658 (2023). 37198043 10.1016/j.pmn.2023.04.007

[19] Avancini, A. et al. Effect of exercise across the head and neck cancer continuum: a systematic review of randomized controlled trials. *Support. Care Cancer*. **31**, 670 (2023). 37924500 10.1007/s00520-023-08126-2 PMC10625510

[20] Samuel, S. R. et al. Effectiveness of exercise-based rehabilitation on functional capacity and quality of life in head and neck cancer patients receiving chemo-radiotherapy. *Support Care Cancer*. **27**, 3913–3920 (2019). 30919154 10.1007/s00520-019-04750-z PMC6728220

[21] D’souza, M., Samuel, S. R. & Saxena, P. P. Effects of exercise training during concomitant chemoradiation therapy in head-and-neck cancer patients: A systematic review. *Indian J. Palliat. Care.* **26**, 531–532 (2020). 33623318 10.4103/IJPC.IJPC_14_20 PMC7888419

[22] Gururaj, R., Samuel, S. R., Kumar, K. V., Nagaraja, R. & Keogh, J. W. L. Effect of exercise based interventions on sleep and circadian rhythm in cancer survivors—a systematic review and meta-analysis. *PeerJ* **12**, e17053 (2024). 38468641 10.7717/peerj.17053 PMC10926908

[23] Soong, R. Y. et al. Exercise Interventions for Depression, Anxiety, and Quality of Life in Older Adults With Cancer: A Systematic Review and Meta-Analysis. *JAMA Netw. Open.* **8**, e2457859 (2025). 39903465 10.1001/jamanetworkopen.2024.57859 PMC11795328

[24] Williams, A. M. et al. Association Between Cognitive Function and Quality of Life in Patients With Head and Neck Cancer. *JAMA Otolaryngol. Head Neck Surg.* **143**, 1228–1235 (2017). 29121151 10.1001/jamaoto.2017.2014 PMC5824302

[25] Von Ah, D. et al. Advanced cognitive training for breast cancer survivors: a randomized controlled trial. *Breast Cancer Res. Treat.* **135**, 799–809 (2012). 22918524 10.1007/s10549-012-2210-6 PMC3677054

[26] Deng, L. et al. The effect of cognitive training on the brain’s local connectivity organization in healthy older adults. *Sci. Rep.* **9**, 9033 (2019). 31227777 10.1038/s41598-019-45463-x PMC6588690

[27] Gavelin, H. M., Lampit, A., Hallock, H., Sabatés, J. & Bahar-Fuchs, A. Cognition-oriented treatments for older adults: A systematic overview of systematic reviews. *Neuropsychol. Rev.* **30**, 167–193 (2020). 32266520 10.1007/s11065-020-09434-8 PMC7305099

[28] Wollesen, B., Wildbredt, A., van Schooten, K. S., Lim, M. L. & Delbaere, K. The effects of cognitive-motor training interventions on executive functions in older people: A systematic review and meta-analysis. *Eur. Rev. Aging Phys. Act.* **17**, 9 (2020). 32636957 10.1186/s11556-020-00240-y PMC7333372

[29] Luebbert, K., Dahme, B. & Hasenbring, M. The effectiveness of relaxation training in reducing treatment-related symptoms and improving emotional adjustment in acute non-surgical cancer treatment: A meta-analytical review. *Psychooncology* **10**, 490–502 (2001). 11747061 10.1002/pon.537

[30] Salsman, J. M. et al. Psychosocial interventions for cancer survivors: A meta-analysis of effects on positive affect. *J. Cancer Surviv.* **13**, 943–955 (2019). 31741250 10.1007/s11764-019-00811-8 PMC7330880

[31] Jagannathan, A. & Juvva, S. Emotions and coping of patients with head and neck cancers after diagnosis: A qualitative content analysis. *J. Postgrad. Med.* **62**, 143–149 (2016). 27320951 10.4103/0022-3859.184273 PMC4970339

[32] Sinha, S. et al. Psycho-oncology/Supportive Care in head–neck cancers patients undergoing radiation therapy: A randomized controlled trial. *South Asian J. Cancer*10.1055/s-0043-1771405 (2023). 41426184 10.1055/s-0043-1771405 PMC12714443

[33] FACT-Cog. *FACIT Group* https://www.facit.org/measures/fact-cog

[34] Oliveira, A. F., Santos, I. M., Fernandes, S., Bem-Haja, P. & Torres, A. Validation study of the Functional Assessment of Cancer Therapy-Cognitive Function-Version 3 for the Portuguese population. *BMC Psychol.* **10**, 305 (2022). 36517827 10.1186/s40359-022-01018-w PMC9748889

[35] Buysse, D. J., Reynolds, C. F., Monk, T. H., Berman, S. R. & Kupfer, D. J. The Pittsburgh Sleep Quality Index: A new instrument for psychiatric practice and research. *Psychiatry Res.* **28**, 193–213 (1989). 2748771 10.1016/0165-1781(89)90047-4

[36] Carpenter, J. S. & Andrykowski, M. A. Psychometric evaluation of the Pittsburgh Sleep Quality Index. *J. Psychosom. Res.* **45**, 5–13 (1998). 9720850 10.1016/s0022-3999(97)00298-5

[37] Löwe, B. et al. A 4-item measure of depression and anxiety: Validation and standardization of the Patient Health Questionnaire-4 (PHQ-4) in the general population. *J. Affect. Disord.* **122**, 86–95 (2010). 19616305 10.1016/j.jad.2009.06.019

[38] Pai, H. D. et al. Translation, cross-cultural adaptation, and validation of the Kannada version of the Exercise Adherence Rating Scale (EARS-Kn) among head and neck cancer (HNC) survivors in a tertiary care setup in India. *Integr. Cancer Ther.* **24**, 15347354251313534 (2025). 39811882 10.1177/15347354251313534 PMC11733881

[39] Hawley-Hague, H., Horne, M., Skelton, D. A. & Todd, C. Review of how we should define (and measure) adherence in studies examining older adults’ participation in exercise classes. *BMJ Open* **6**, e011560 (2016). 27338884 10.1136/bmjopen-2016-011560 PMC4932302

[40] Adzrago, D., Walker, T. J. & Williams, F. Reliability and validity of the Patient Health Questionnaire-4 scale and its subscales of depression and anxiety among US adults based on nativity. *BMC Psychiatry* **24**, 213 (2024). 38500115 10.1186/s12888-024-05665-8 PMC10949792

[41] Meneses-Echávez, J. F., González-Jiménez, E. & Ramírez-Vélez, R. Effects of supervised multimodal exercise interventions on cancer-related fatigue: Systematic review and meta-analysis of randomized controlled trials. *BioMed. Res. Int.* **2015**, 328636 (2015). 26167483 10.1155/2015/328636 PMC4488083

[42] Myers, J. S., Erickson, K. I., Sereika, S. M. & Bender, C. M. Exercise as an intervention to mitigate decreased cognitive function from cancer and cancer treatment: An integrative review. *Cancer Nurs.* **41**, 327–343 (2018). 29194066 10.1097/NCC.0000000000000549 PMC5975081

[43] Pinheiro, V. H. G., Pinto, S. S., Cardozo, P. S., Andrade, L. S. & Alberton, C. L. The effect of exercise on cognitive function in breast cancer survivors: Systematic review and meta-analysis of randomized controlled trials. *Support Care Cancer* **33**, 577 (2025). 40514607 10.1007/s00520-025-09647-8

[44] Gothe, N. P., Erlenbach, E. & Salerno, E. A. Yoga improves self-reported cognitive function among cancer survivors: Results from the STAYFit trial. *Front. Cognit.*10.3389/fcogn.2024.1334727 (2024). 42339439 10.3389/fcogn.2024.1334727 PMC13281268

[45] Cheung, Y. T. et al. Minimal clinically important difference (MCID) for the Functional Assessment of Cancer Therapy: Cognitive function (FACT-Cog) in breast cancer patients. *J. Clin. Epidemiol.* **67**, 811–820 (2014). 24656406 10.1016/j.jclinepi.2013.12.011

[46] Maqbali, M. A., Sinani, M. A., Alsayed, A. & Gleason, A. M. Prevalence of sleep disturbance in patients with cancer: A systematic review and meta-analysis. *Clin. Nurs. Res.* **31**, 1107 (2022). 35484919 10.1177/10547738221092146 PMC9266067

[47] Rolke, H. B., Bakke, P. S. & Gallefoss, F. HRQoL changes, mood disorders and satisfaction after treatment in an unselected population of patients with lung cancer. *Clin. Respir. J.* **4**, 168–175 (2010). 20565496 10.1111/j.1752-699X.2009.00171.x

[48] Palesh, O. G. et al. Prevalence, demographics, and psychological associations of sleep disruption in patients with cancer: University of Rochester Cancer Center–Community Clinical Oncology Program. *JCO* **28**, 292–298 (2010). 10.1200/JCO.2009.22.5011 PMC2815717 19933917

[49] Beck, S. L., Schwartz, A. L., Towsley, G., Dudley, W. & Barsevick, A. Psychometric evaluation of the Pittsburgh sleep quality index in cancer patients. *J. Pain Symptom Manage.* **27**, 140–148 (2004). 15157038 10.1016/j.jpainsymman.2003.12.002

[50] Pai, A., Sivanandh, B. & Udupa, K. Quality of sleep in patients with cancer: A cross-sectional observational study. *Indian J. Palliat. Care* **26**, 9–12 (2020). 32132776 10.4103/IJPC.IJPC_164_19 PMC7017701

[51] So, W. K. W. et al. Quality of life in head and neck cancer survivors at 1 year after treatment: The mediating role of unmet supportive care needs. *Support. Care Cancer.* **22**, 2917–2926 (2014). 24839941 10.1007/s00520-014-2278-0

[52] Davidson, A. & Williams, J. Factors affecting quality of life in patients experiencing facial disfigurement due to surgery for head and neck cancer. *Br. J. Nurs.* **28**, 180–184 (2019). 30746969 10.12968/bjon.2019.28.3.180

[53] Moore, K. A., Ford, P. J. & Farah, C. S. ‘I have quality of life…but…’: Exploring support needs important to quality of life in head and neck cancer. *Eur. J. Oncol. Nurs.* **18**, 192–200 (2014). 24238663 10.1016/j.ejon.2013.10.010

[54] Zhou, X., Kong, Y., Yu, B., Shi, S. & He, H. Effects of exercise on sleep quality in general population: Meta-analysis and systematic review. *Sleep Med.* **125**, 1–13 (2025). 39556996 10.1016/j.sleep.2024.10.036

